# Estimating worldwide effects of non-pharmaceutical interventions on COVID-19 incidence and population mobility patterns using a multiple-event study

**DOI:** 10.1038/s41598-021-81442-x

**Published:** 2021-01-21

**Authors:** Nikolaos Askitas, Konstantinos Tatsiramos, Bertrand Verheyden

**Affiliations:** 1 IDSC - Research Data Center, IZA - Institute of Labor Economics, Bonn, 53113 Germany; 2 Department of Economics and Management, University of Luxembourg, 1359 Luxembourg, Luxembourg; 3 Luxembourg Institute of Socio-Economic Research (LISER), Labour Market, 4366 Luxembourg, Luxembourg

**Keywords:** Diseases, Infectious diseases, Viral infection

## Abstract

Various non-pharmaceutical interventions were adopted by countries worldwide in the fight against the COVID-19 pandemic with adverse socioeconomic side effects, which raises the question about their differential effectiveness. We estimate the average dynamic effect of each intervention on the incidence of COVID-19 and on people’s whereabouts by developing a statistical model that accounts for the contemporaneous adoption of multiple interventions. Using daily data from 175 countries, we show that, even after controlling for other concurrent lockdown policies, cancelling *public events*, imposing restrictions on *private gatherings* and closing *schools* and *workplaces* had significant effects on reducing COVID-19 infections. Restrictions on *internal movement* and *public transport* had no effects because the aforementioned policies, imposed earlier on average, had already de facto reduced human mobility. *International travel* restrictions, although imposed early, had a short-lived effect failing to prevent the epidemic from turning into a pandemic because they were less stringent. We interpret the impact of each intervention on containing the pandemic using a conceptual framework which relies on their effects on human mobility behaviors in a manner consistent with time-use and epidemiological factors.

## Introduction

In December 2019, the COVID-19 outbreak was first registered in Wuhan China. The World Health Organization declared it a “Public Health Emergency of International Concern” on January 30, 2020 and escalated it to a pandemic on March 11, 2020. The disease has been recorded in over 200 countries and territories with several millions of confirmed cases and a case fatality rate which depicted its first peak on April 30, 2020 at over seven percent^1^. In most countries, a number of non-pharmaceutical interventions (henceforth referred to as NPIs, or lockdown policies) were undertaken to achieve “social distancing” as a means of blocking the main transmission routes of the virus^2^. The aim of these interventions was to slow down the pandemic by restricting mobility, and thus to remain within the capacity of the health systems^3–5^. Understanding the effectiveness of these policies and their channels is important, as policy makers and the society at large seek to achieve an optimal health outcome in the fight against the pandemic at the lowest economic cost.

We study whether and to what extent each of the adopted lockdown policies affect the daily *incidence* of COVID-19, using coded government response data obtained from the Oxford COVID-19 Government Response Tracker (OxCGRT) and epidemiological data from the European Centre for Disease Prevention and Control (EDCD). We also study the channels of these effects by analyzing the impact of each of the lockdown policies on *population mobility patterns* using data from Google Community Mobility Reports.

The main contributions of the paper are the following. First, we develop a statistical model that provides non-parametric estimates for the effect of each intervention over time accounting for multiple contemporaneous interventions. The model also includes i) country fixed effects adjusting for time-invariant differences across countries such as population density, age distribution, per-capita income, health systems, quality of institutions and social norms, ii) non-parametric controls for time trends since the first-observed COVID-19 case, iii) day-of-week fixed effects, iv) the stock of infectious people and v) daily weather conditions. We show that ignoring the influence of confounding policies leads to biased estimates by attributing the effect of other contemporaneous interventions on COVID-19 incidence and mobility patterns to the intervention of interest.

Second, we exploit within and between country variation, across 175 countries, in the level of intensity (stringency and geographic scope), as well as the timing, of a wide range of interventions such as: i) *international travel* controls, ii) *public transport* closures, iii) cancelation of *public events*, iv) restrictions on *private gatherings*, v) *school* closures, vi) *workplace* closures, vii) *stay-at-home* requirements and viii) *internal mobility* restrictions (across cities and regions).

Third, beyond COVID-19 incidence, we study the impact of lockdown policies on mobility patterns. Such patterns capture the time spent in a number of types of places such as: i) *retail and recreation*, ii) *grocery and pharmacy*, iii) *parks*, iv) *transit stations*, v) *workplaces* and vi) *residential areas*. In addition to being intricately related to the economy^6^, each of these types of places is characterized by different epidemiologically relevant features and, therefore, has a different potential for viral transmission. The mobility data can then be viewed as a measure of compliance to the policies introduced, and a mediator between policies and the spread of the disease, providing a comprehensive and consistent account of the policy response to the pandemic.

Our work aims at establishing empirically which among the many interventions that have been implemented to fight the first wave of the pandemic are on average the most effective. This knowledge can serve as a benchmark for future waves of the COVID-19 or future pandemics^7,8^. To the best of our knowledge, there is no other study estimating the independent effects of several lockdown policies on both the incidence of COVID-19 and on mobility patterns within a framework that accounts for concurrent interventions using such a large cross section. Studies investigating the effect of lockdown policies on the incidence of COVID-19 have either a regional—within country - focus^9–15^ or are international but relying either on a limited cross section^16,17^, or on a larger cross-section but using a one-dimensional measure of social distancing, which does not allow assessing the independent effects of different types of policies^18^.

The rest of the paper is structured as follows. “Data” Section presents the data and “Model” section describes the model and identification issues. “Results” section presents results and robustness analysis. “Discussion” section summarizes the findings, including a discussion linking the evidence on COVID-19 incidence with mobility patterns, and discusses future research.

## Data

We use publicly available data on several NPIs across 175 countries, collected by the OxCGRT, which include their date of introduction as well as qualitative time-varying information on their intensity^19^. We focus on the following interventions: i) *international travel controls*, ii) *closure of public transport*, iii) *cancelation of public events*, iv) *restrictions on private gatherings*, v) *closure of schools*, vi) *closure of workplaces*, viii) *restrictions on internal movement* and viii) *stay-at-home requirements*.

We construct an intensity measure for each policy on a scale from 1 to 6, which combines its stringency and geographic scope. Stringency can have three levels depending on whether the policy is: (i) recommended, (ii) mandatory with some flexibility, or (iii) mandatory with no flexibility. Geographic scope can have two levels depending on whether the policy is: (i) geographically targeted, or (ii) applied to the entire country. Recommended, but geographically targeted policies, obtain an intensity value of 1, while mandatory policies with no flexibility applied to the entire country obtain an intensity value of 6. For the remaining combinations of stringency and geographic scope, the intensity value is higher for each level of stringency when the policy is applied to the entire country. For example, when the schooling policy receives an intensity score of 4, it means that it was not made mandatory in all schools or in all education levels, but it was applied to the entire country. A score of 5 means that it was made mandatory to all schools and education levels, but only in some areas of the country. A score of 6 means that it is mandatory for all schools and areas of the country. The average intensity level across countries, at policy implementation day, is 2.9 for international travel controls, 3.8 for public transport closures, 5.4 for school closures, 3.8 for workplace closures, 4.7 for canceling public events, 4 for restrictions on private gatherings, 3.2 for stay-at-home requirements and 4.2 for internal movement restrictions.

Figure A1 in the Supplementary Information presents the distribution of the number of days it took for each policy to be introduced after the first COVID-19 case averaged across countries. The distribution for the international travel controls is bimodal with the first mode well ahead of the first case. All policies have a main mode close to zero, with cancellation of public events and school closures enacted earlier, followed by restrictions on private gatherings and workplace closures, stay-at-home requirements, internal mobility restrictions and public transport restrictions, and a secondary mode late into the epidemic.

The data on daily confirmed COVID-19 cases is obtained from the ECDC for each country, which examines reports from health authorities worldwide in a systematic way to produce daily statistics on COVID-19^20^. The observed variation of the daily incidence of COVID-19 cases may contain random reporting imperfections. To remove such random variation from the data, we use a 3-day moving average in the analysis of the confirmed new cases and the inverse hyperbolic sine transformation^21^, in order to include days without new confirmed cases.

To study how mobility patterns have evolved worldwide, we resort to Google’s Community Mobility Reports^22^. Google creates these mobility data with *“aggregated, anonymized sets of data from users who have turned on the Location History setting”* on their phone. They express the percentage of deviation from a baseline of the *“number of visits and length of stay”* at a number of different types of places. The baseline is the median value for the corresponding day of the week during the 5-week period from January 3, 2020 to February 6, 2020. These data contain information on various epidemiologically relevant categories of places such as i) *retail and recreation* covering visits to restaurants, cafes, shopping centers, theme parks, museums, libraries, and movie theaters; ii) *grocery and pharmacy* covering grocery markets, food warehouses, farmers markets, specialty food shops, drug stores, and pharmacies; iii) *parks* encompassing national parks, public beaches, marinas, dog parks, plazas, and public gardens; iv) *transit stations* covering subway, bus and train stations, v) *workplaces* and vi) *residential areas*. Information on mobility patterns is available for 123 countries.

Figure A2 in the Supplementary Information presents the distribution of these mobility data averaged across countries both before and after the first confirmed COVID-19 case. Before the first case, all distributions are highly concentrated around zero, which suggests no substantial change of movement compared to the baseline period (January 3, 2020 to February 6, 2020). After the first COVID-19 case, retail and recreation as well as transit stations have a mean of just above -40, suggesting a 40 percentage points drop, while differing in their variance. Grocery and pharmacy as well as workplaces have a mean of around -20 differing in their skewness (grocery and pharmacy is heavy on the left). Parks stand out for having a mean closest to zero and being heavy on the right. Finally, residential areas have a mean of just over 10. In term of densities, retail and recreation, grocery and pharmacy, transit stations as well as workplaces are somewhat similar, while parks and residential areas are on their own on opposite sides.

A limitation of these data is related to Google’s ability to accurately locate phones and to correctly categorize places, which varies both across countries as well as within (urban vs. rural areas). Although not perfectly, our model, which is presented in the next Section, can account for these limitations by controlling for time-invariant country differences through a set of country-specific dummies.

## Model

We follow an event-study approach around the time of policy implementation, which we extend to account for multiple events. The single-event model (e.g.,^23^) can be expressed with the following equation:

1 $$\begin{aligned} Y_{c,t}= \displaystyle {\sum _{j > -r}} \alpha _{j} I[j=t-t_c^{\pi }] +\displaystyle {\sum _{l}} \gamma _l I[l=t] + \displaystyle {\sum _{d}}\delta _d I[d_c(t) = d]+ \theta _c + \phi Z_{c,t} + \epsilon _{c,t}, \end{aligned}$$

where $Y_{c,t}$ denotes the outcome in country *c* at event time *t*, which is either the natural logarithm of daily incidence (new cases), or the percent of deviation of various population mobility types from a pre-pandemic baseline (the median value for the corresponding day of the week during the 5-week period from January 3, 2020 to February 6, 2020).

The first term, on the right-hand side, is a set of event time dummies for the intervention of interest $\pi$, where $t_c^{\pi }$ denotes the implementation time of policy $\pi$ in country *c*. These event time dummies take the value one at event time *j*, and zero otherwise. The event time coefficients of interest $\alpha _{j}$ measure the impact of intervention $\pi$ at time *j*, relative to the reference period in the pre-intervention period. The second term, on the right- hand side of Eq. (1), is a set of dummies controlling non-parametrically for trends in the time since the first-observed COVID-19 case. The third term is a set of day-of-the-week dummies controlling for potential day-specific differences both in terms of reporting of new cases and of mobility patterns (here $d_c(t)$ returns the day of the week for event time *t* in country *c*). The fourth term represents country fixed effects. Country fixed effects capture time-invariant characteristics such as population density, age distribution, quality of institutions, health systems, social norms, and other epidemiologically relevant factors. They also account for differences in the way countries report COVID-19 incidence and differences in Google’s ability to geo-locate phones and detect types of places across countries. The fifth term contains country-specific, time-varying effects, such as the 14-day moving averages of temperature and precipitation at the capital city of country *c* at $t-7$, as well as the sum of new cases in the last twenty days minus the sum of deaths in the same time period in country *c* at time $t-2$. By including these controls, we account for weather conditions—affecting both the virus and population behavior—and we capture the size of the pool of infectious people, which is a crucial factor both when the outcome is the daily incidence of COVID-19, in line with the SIR framework^24^, as well as when the outcome is mobility patterns, as populations may react to the perceived threat of contamination. The last term is the error term. We cluster standard errors at the country level.

We restrict the sample to the set of countries that acted before the first 300 COVID-19 cases, i.e., countries which acted around the same part of the pandemic curve. This restriction is important because for countries that acted in the early phase of the pandemic, it is reasonable to assume that the variation in the intensity of the lockdown policies is not correlated with the outcomes of interest, but rather it is driven by other exogenous country-specific factors. This strengthens the identification of the policy effects because it excludes the possibility that the intensity of the policy is related to the stage of the pandemic (reverse causality). This sample restriction also helps to establish that the parallel trend assumption in event study designs holds, which in our setting requires that countries applying different policies at different intensities were following similar trends in COVID-19 incidence prior to their introduction. Finally, in order to address well known under-identification issues^25,26^, which arise due to the inclusion of time and country fixed effects, in addition to the event time effects, we use several event times as reference. In particular, while the event-time window runs from [$-45, +45$] days around the day of the policy implementation, we indicate in the first term of Eq. (1) with $j>-r$ that we drop a number of event time dummies before $-r$. We limit $r=10$ for COVID-19 incidence and $r=20$ for the mobility outcomes. When we study COVID-19 incidence we set *r* equal to 10 because the epidemic curve is still in its early phase. We set *r* equal to 20 for mobility because mobility data are available for a longer time period.

When evaluating the effect of the intervention of interest $\pi$, it is important to take into account the presence of other contemporaneous interventions, which can have their own contribution in affecting the outcome, and thus, if ignored can lead to biased estimates. However, identifying the effect of the policy of interest $\pi$ with multiple events is more challenging than in the single-event case, especially when the multiple events fully overlap during the event window of the policy of interest. Concurrent NPIs, denoted by $\pi '$, can be controlled for by introducing in Eq. (1) a new term, $F^{\pi '}[j=t-t_c^{\pi }]$, which is a set of dummies—one dummy for each event time of the policy of interest $\pi$—which are equal to one if any other interventions $\pi '$ are in effect at event time *j* for country *c*. The multiple-event regression equation can then be written as follows:

2 $$\begin{aligned} Y_{c,t}= \displaystyle {\sum _{j > -r}} \alpha _{j} I[j=t-t_c^{\pi }] +\displaystyle {\sum _{j}}\beta _j F^{\pi '}[j=t-t_c^{\pi }] + \displaystyle {\sum _{l}} \gamma _l I[l=t] + \displaystyle {\sum _{d}}\delta _d I[d_c(t) = d]+ \theta _c + \phi Z_{c,t} + \epsilon _{c,t}, \end{aligned}$$

The identification problem in the multiple-event case emerges as soon as other policies have been introduced before the start of the event window of policy ${\pi }$. This would result in a complete overlap of policies within the event window, making it impossible to separately identify the effect of the event of interest from the other contemporaneous events. When other policies are enacted within the event window, then the two sets of event dummies are not perfectly collinear so the coefficient estimates $\alpha _j$ and $\beta _j$ can be separately identified, but at the cost of high variance because of multicollinearity.

To achieve identification in the multiple-event case, we use the value of intensity of each policy, which varies over time and across policies within countries, as well as across countries. This variation of policy intensity permits identification of the effect of the policy of interest $\pi$, while taking into account concurrent NPIs, $\pi '$. The extended multiple-event regression equation can be written as follows:

3 $$\begin{aligned} Y_{c,t}= \displaystyle {\sum _{j > -r}} \alpha _{j} S^{\pi }[j=t-t_c^{\pi }] +\displaystyle {\sum _{j}}\beta _j \bar{S}^{\pi '}[j=t-t_c^{\pi }] + \displaystyle {\sum _{l}} \gamma _l I[l=t] + \displaystyle {\sum _{d}}\delta _d I[d_c(t) = d]+ \theta _c + \phi Z_{c,t} + \epsilon _{c,t}, \end{aligned}$$

where the first term, $S^{\pi }[j=t-t_c^{\pi }]$, is equal to the value of intensity of the policy of interest $\pi$ in country *c* at event time *j*, and zero otherwise. The second term, $\bar{S}^{\pi '}[j=t-t_c^{\pi }]$, is equal to the average value of intensity of all other contemporaneous policies $\pi '$ of country *c* at the event time *j*, and zero if there are no other policies active at that event time. That is, we extend Eq. (2) in two ways: 1) we multiply the event dummies for policy $\pi$ with the intensity value of the policy at event time *j*—in other words, $I[j=t-t_c^{\pi }]$ in Eq. (2) generalizes to $S^{\pi }[j=t-t_c^{\pi }]$ in Eq. (3); and 2) we multiply the dummies controlling for the presence of any other policies $\pi '$—at event time *j* for policy $\pi$—with their average intensity value at event time *j*—in other words, $F^{\pi '}$ in Eq. (2) generalizes to $\bar{S}^{\pi '}_{c,t}$ in Eq. (3).

As discussed above, identification relies on the plausibly exogenous variation in the timing and intensity of various interventions among the set of countries which acted during the early phase of the pandemic, conditional on the stock of infectious people, time effects since the first-observed case, day-of-the-week effects, country fixed effects and weather conditions. This variation makes it possible to separately identify the effect of intervention $\pi$ from that of other concurrent NPIs, $\pi '$. The coefficients $\alpha _j$ in Eq. (3) measure the effect of a unit change in the value of intensity $\pi$ at event time *j* on the outcome of interest. For estimating Eq. (3), we use the ordinary least squares linear regression method with many levels of fixed effects^27^. All estimated regressions use the maximum sample of countries for which each policy has been implemented.

## Results

“Lockdown policies on COVID-19 incidence” section presents the results for the effects of lockdown policies on the incidence of COVID-19, “Robustness” section discusses robustness checks and “Lockdown policies on google mobility patterns” section presents the effects of the lockdown policies on population mobility patterns.

### Lockdown policies on COVID-19 incidence

**Figure 1:**
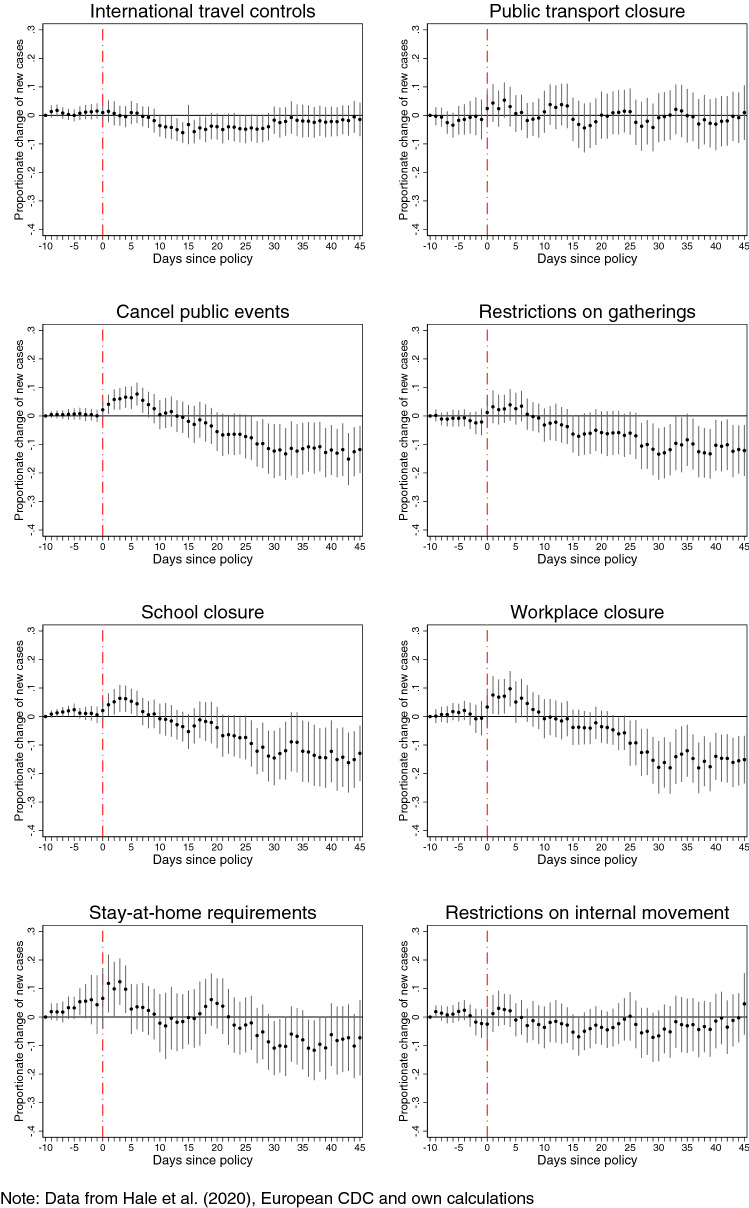
Effects of lockdown policies on **COVID-19** confirmed new cases (3-day moving average, in logs) with controls for concurrent policies.

We report the estimates of the dynamic effect of each intervention on COVID-19 incidence (logarithmic daily confirmed new cases) in Fig. 1. These estimates correspond to the parameters of the first term of Eq. (3) and measure the percentage change in COVID-19 incidence for a unit change in the value of intensity of the policy of interest, compared to the reference pre-intervention period, which starts 10 days before the intervention.

We find that the most effective interventions in containing the spread of COVID-19 are those aiming at reducing contacts in large groups, such as canceling of *public events* and restrictions on *private gatherings*, or reducing contacts with high frequency, such as *school* and *workplace* closures. We observe a drop in the incidence of COVID-19 starting about one week after these four policies were implemented, which becomes negative and significantly different from zero (at the 5% significance level) in the next two weeks. Compared to the reference pre-intervention period, a unit increase in the value of intensity of canceling of *public events* or restrictions on *private gatherings* leads to a decrease of about 12% in the number of daily infections 6 weeks after the intervention was implemented. For *school* and *workplace* closures, the corresponding effect is around 12% and 15%, respectively.

*Stay-at-home requirements*, generally introduced as a last resort, take more time to bring incidence below the reference period, and when they do, their effect becomes negative and significantly different from zero over a limited number of days.

*International travel controls* become effective at reducing incidence about 10 days after their introduction, for a duration of about two and a half weeks, after which they cease to be effective. Our finding of a short-lived effect of international travel controls is consistent with available evidence^13^ which shows that reducing air traffic flows to and from China led to a slowdown of the spread of the epidemic across countries but does so in an early and shorter time frame than our study. Had international travel controls been more stringent early on, they would have probably prevented the epidemic from turning into a pandemic. Unfortunately, these controls were applied on average at the lowest intensity of all policies and they hence became as suitable a policy against a pandemic as a broken levee is against a flood.

Restrictions on *internal movement* and *public transport* closures have a negligible impact over the entire event-time window. That public transport restrictions were not effective is explained by the earlier introduction of other types of restrictions, which lowered the use of public transport and de facto reduced internal movement. Once the policies that restrict the activities causing most of public transportation have been imposed (e.g., workplace and school closures, canceling of public events and restrictions on private gatherings), restrictions on public transport only reduce the mobility of a limited number of remaining users. Therefore, the net impact of public transport restrictions on COVID-19 incidence is limited because preceding policies had already substantially reduced the risk of infections in such places. A similar logic applies to explain the weak effect of internal mobility restrictions.

Finally, we compare the estimates between two versions of the model, with and without controls for concurrent policies, that is, we estimate Eq. (3) with or without the second term. We find that the estimated effects of the lockdown interventions without controlling for concurrent interventions are biased (reported in Figure A3 in Supplementary Information), suggesting that all policies are almost equally effective in reducing the incidence of COVID-19. It is only once we control for confounding due to the contemporaneous presence of other interventions that we find differences in the effectiveness of each policy, which are masked when all policies are either treated separately or as a group. These findings are consistent with and in fact extend studies estimating the joint effect of a group of policies (e.g.,^14,15^) by identifying the separate effect of each of them.

### Robustness

The results on COVID-19 incidence are robust to a battery of checks, such as perturbing the length of the windows used for the moving averages of new cases (from 3 to 7 days), or instead using the raw daily new cases, and perturbing the lags of various covariates, such as weather conditions and the pool of the infectious population. In order to deal with multicollinearity, the baseline model controls for concurrent policies by aggregating them and exploiting their mean value of intensity. Results are qualitatively unchanged when this level of aggregation is reduced by splitting the concurrent policy controls into two groups (mobility and non-mobility related). They are also robust to controlling for the policy profile, which captures the complete set of policies in force at each event time. Finally, they are robust to enlarging the sample to the full set of countries, which includes those which reacted after the first 300 confirmed COVID-19 cases, at the cost of losing the parallel trend in the pre-intervention period.

### Lockdown policies on google mobility patterns

**Figure 2:**
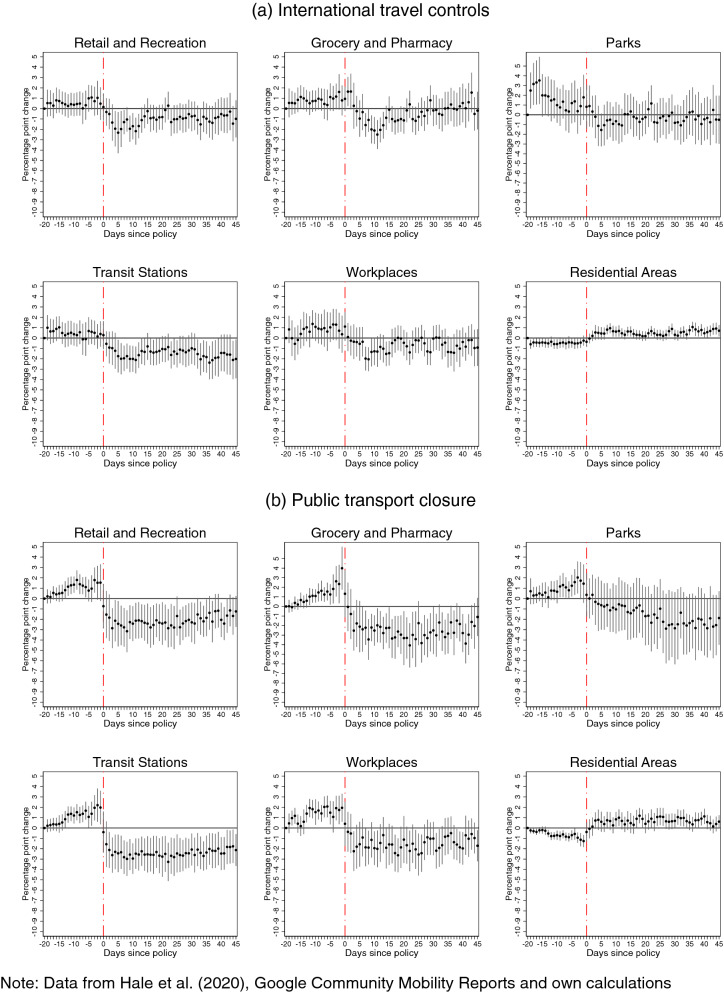
Effects of **international travel controls** (panel **a**) and **closure of public transportation** (panel **b**) on Google mobility patterns.

**Figure 3:**
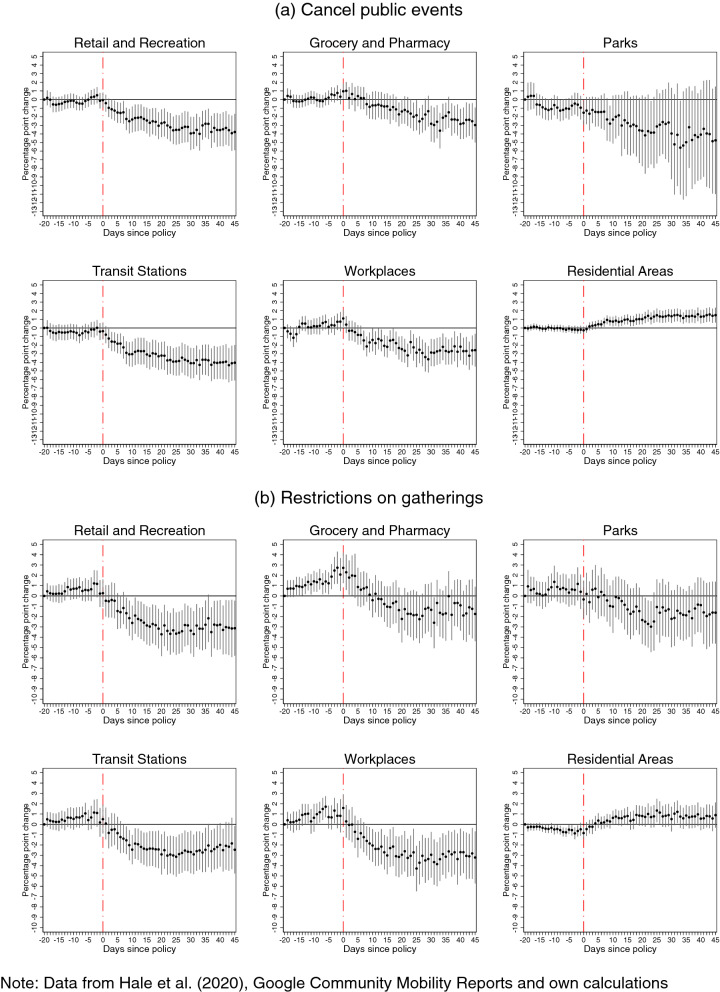
Effects of **public events cancellations** (panel **a**) and **restrictions on gatherings** (panel **b**) on Google mobility patterns.

**Figure 4:**
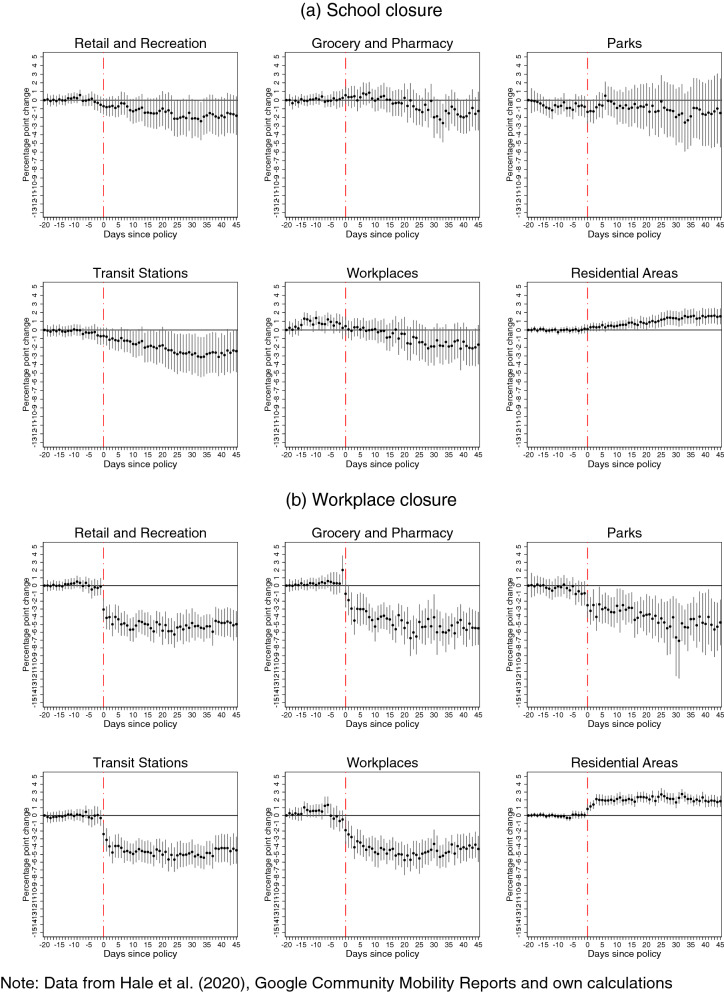
Effects of **school** (panel **a**) and **workplace** (panel **b**) closures on Google mobility patterns.

**Figure 5:**
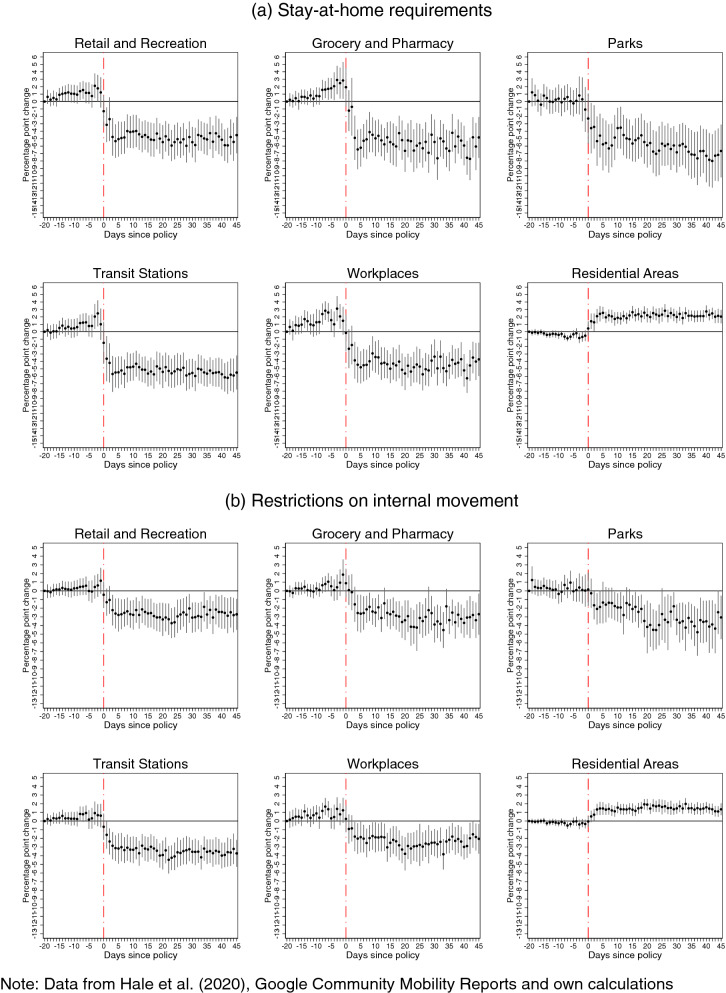
Effects of **stay-at-home requirements** (panel **a**) and **restrictions on internal mobility** (panel **b**) on Google mobility patterns.

Google mobility patterns are observed as percentage point deviations from a reference calendar period before the onset of COVID-19, which is the median value for the corresponding day of the week during the 5-week period from January 3, 2020 to February 6, 2020. The coefficient estimates of interest—first term of Eq. (3)—measure the percentage point change in mobility patterns for a unit change in the value of intensity of each intervention, compared to the reference pre-intervention period which starts 20 days before the intervention. Similar to the analysis on COVID-19 incidence, we obtain estimates both with and without controls for concurrent interventions. The estimates with controls for concurrent policies are presented in Figs. 2, 3, 4 and 5, while those without controlling for confounding policies can be found in Figs. A4, A5, A6 and A7 in the Supplementary Information A.

Without controlling for confounding policies, our estimations show negative effects of each intervention on several patterns of mobility outside the residence, and positive effects on the amount of time spent in the place of residence. In many cases, this decline starts even before imposing the restrictions (which is suggestive of anticipation) or fades out in the post-intervention period. However, once we control for concurrent policies, a more nuanced picture emerges with subtle but significant differences: we find evidence of a reversal in the mobility patterns before imposing the restrictions, and that many of the policy effects in the post-intervention period are more persistent, but smaller in magnitudes, or sometimes not significantly different from zero. The differences observed between these sets of estimates provide strong evidence that, similar to the analysis of COVID-19 incidence, not accounting for concurrent policies leads to biased estimates of how each policy affected the time spent at various places.

Furthermore, eliminating the bias, which stems from confounding policies, provides evidence that it is policies affecting mobility patterns and not policies ex-post responding to de facto changing mobility patterns in the population. It also reveals a spike in movements to groceries and pharmacies prior to the introductions of several NPIs, such as public transport and workplace closure, as well as stay-at-home and internal movement restrictions. This is consistent with the widely reported runs on the shelves in anticipation of lockdowns, concerns about imminent shortages, as well as with inadvertent signaling from these interventions about the threat-level of the pandemic. This increase in mobility to grocery stores and pharmacies, in the days preceding the lockdowns, may in part explain the peak in incidence in the days that followed the introduction of most policies. Finally, the persistence of the effect on mobility patterns suggests that compliance does not decline over time, at least within the 45-day window of our study.

Focusing on the estimates which control for concurrent policies, we find that the cancellation of *public events* and restrictions on *private gatherings*, which aim at restricting interactions in large groups that are difficult to control and track, have large and persistent negative impacts on retail and recreation, transit stations, workplaces and to a lesser extent groceries and pharmacies (Fig. 3), whose magnitude is around 3-4 percentage points per unit value of policy intensity.

For *school* and *workplace* closures, we find that they reduce the time spent to activities undertaken outside the residential area (Fig.  4). The magnitude of these effects is much larger for workplace than for school closures, with a unit increase in the value of intensity of the policy leading to a decline of up to about 5 and 1-2 percentage points, respectively. This difference in the magnitudes is consistent with the following observations. First, working adults are more likely to make use of more means of transport while commuting to work than pupils attending school. Second, while pupils staying at home might constrain the mobility of one parent, closing workplaces affects the mobility of all working adults in the household.

For *stay-at-home* requirements, we find large effects in all population mobility patterns (panel (a) of Fig. 5), which are similar in magnitude to those found for workplace closures. Similarly, *internal mobility* restrictions have significant effects on all mobility patterns (panel (b) of Fig. 5), although with about half the magnitude of the effects of stay-at-home requirements.

This evidence suggests that several policies such as closing workplaces, canceling public events and restricting private gatherings generate spillover effects on other activities such as using public transport, which is consistent with and extends available evidence^28^ on how the numerosity of public transport and shared mobility services was reduced during the pandemic as people preferred private vehicles or bicycles. Taking these spillover effects into account, it is natural that *public transport* closures have a smaller impact on all mobility types (estimates reported in Fig. 2). Finally, quite unsurprisingly, we also find a small impact on mobility from *international travel* controls.

## Discussion

In this paper, we developed a multiple-event model to study the causal effect of eight lockdown policies on the daily incidence of COVID-19 and on population mobility patterns across 175 countries, exploiting variation in the timing and intensity of these confinement policies. The key contribution of the paper is that we modeled the dynamic effects of each policy, while controlling for the dynamic impact of concurrent interventions, and showed that ignoring their presence leads to biased estimates.

Our findings establish that canceling public events and enforcing restrictions on private gatherings, as well as closing schools and workplaces, had the largest effects on curbing the pandemic. These four policies led to large and statistically significant declines in the incidence of COVID-19. Stay-at-home requirements were used as a policy of last resort and helped to slow down the growth of daily incidence. International travel controls had an early and short-lived effect on the incidence of COVID-19. Finally, restrictions on internal movement and public transport closures did not lead to decreases in the COVID-19 incidence. This latter finding does not imply that restricting internal movement or public transport is epidemiologically irrelevant. It rather suggests that once policies such as workplace and school closures, canceling of public events and restrictions on private gatherings -that restrict the activities which generate most of public transportation and internal mobility- have been imposed, the remaining travelers are so few and so sparsely distributed that the probability of infection during travel is low.

It is worth noting that these estimates refer to an average effect of lockdown policies on COVID-19 incidence across countries, without excluding the possibility of heterogeneous effects in single cases in which these policies had been implemented following a different timing. Such heterogeneity analysis is beyond the scope of this study.

To understand the impact of each policy on containing the pandemic, three remarks are in order. First, each policy delivers its effect against the pandemic by changing people’s whereabouts in order to reduce contagion. This effect is delivered both directly to the place or type of behavior the policy targets (e.g., closing workplaces directly targets the workplace) as well as indirectly by affecting additional places and behaviors. For example, preventing people from going to work causes them to stay at home longer, e.g., telecommuting or being unemployed, but also reduces their use of public transport and changes their consumption habits. Second, the potential for viral transmission of each place or type of behavior depends on several of its epidemiologically relevant characteristics, the most important of which are: i) numerosity, ii) density, iii) social norms, iv) geographical range, v) tracking ability and vi) frequency. Third, places and behaviors also differ from a time-use perspective with relatively more time spent at home compared to activities outside the residential area.

Cancellation of *public events* and restrictions on *private gatherings* contributed to reducing COVID-19 incidence by preventing exposure to numerous and dense locations, where the two-meter social-distancing rule is more likely to be violated and contact tracing is difficult. Also, in these locations, there are norms of accepted behavior which are also likely to violate social distancing rules and in addition can have a large epidemiological range within and across countries (e.g., soccer games). *Workplace* and *school* closures were also effective by reducing activities at locations which are less dense and less populous than public events and private gatherings, as well as easier to track, but they have a much higher frequency. *Stay-at-home* requirements contributed to reducing COVID-19 incidence by increasing the time spent at home, while restrictions on *internal movements*, *public transport* closures and *international travel* controls reduced various types of commuting. Travel controls failed to prevent the pandemic despite some early and short-lived effect, much like a weak levee fails to prevent a flood, because they were implemented early but with the lowest mean intensity value among the eight policies we consider in this study. Restrictions on internal movement and public transport were not as effective in reducing the incidence of COVID-19 because of the spillover effect on mobility of other interventions imposed earlier (Figure A2 in Supplementary Information), such as workplace closures and cancellations of public events and private gatherings. As a result, when these restrictions were introduced, their net effect on reducing infections was limited because the remaining risk of infections was already low.

The empirical framework proposed in this study is suitable for estimating dynamic effects with multiple events, which can be applied in many settings. In the case of COVID-19, it can be used to investigate whether and to what extent there are differences in how lockdown policies affect COVID-19 cases relative to the way they affect deaths, the role of masks, which is also the subject of research^29^, as more publicly available data across countries are becoming available allowing to test their effectiveness once other contemporaneous interventions are controlled for, as well as the impact of the exit strategies from the lockdowns on the dynamics of the pandemic.

## Supplementary Information

Supplementary Figures.

## Data Availability

The datasets generated during and/or analysed during the current study are available from the corresponding author on reasonable request.

## Code availability

The code implementing the model is available from the corresponding author on reasonable request. Code Availability: The code implementing the model is available from the corresponding author on reasonable request.

## References

[1] Johns Hopkins Coronavirus Resource Center CSSE, COVID-19 Dashboard. https://coronavirus.jhu.edu/map.html (2020).

[2] Guo Z (2020). Aerosol and surface distribution of severe acute respiratory syndrome coronavirus 2 in hospital wards, Wuhan, China, 2020. Emerg. Infect. Dis..

[3] Stang A, Stang M, Jöckel K (2020). Estimated use of intensive care beds due to covid-19 in Germany over time. Dtsch. Arztebl. Int..

[4] United States Centers for Disease Control and Prevention, COVID-19 Module Data Dashboard—Patient Impact and Hospital Capacity Pathway. https://www.cdc.gov/nhsn/covid19/report-patient-impact.html (2020).

[5] Moghadas, S. M. *et al.* Projecting hospital utilization during the covid-19 outbreaks in the United States. In *Proceedings of the National Academy of Sciences* **117**, 9122–9126, 10.1073/pnas.2004064117 (2020). https://www.pnas.org/content/117/16/9122.full.pdf. 10.1073/pnas.2004064117 PMC7183199 32245814

[6] Bonaccorsi G (2020). Economic and social consequences of human mobility restrictions under COVID-19. Proc. Natl. Acad. Sci..

[7] Siegenfeld AF, Taleb NN, Bar-Yam Y (2020). Opinion: what models can and cannot tell us about covid-19. Proc. Natl. Acad. Sci..

[8] Lazarus J. e. a (2020). Keeping governments accountable: the COVID-19 Assessment Scorecard (COVID-SCORE). Nat. Med..

[9] Badr, H. S. *et al.* Association between mobility patterns and covid-19 transmission in the usa: a mathematical modelling study. *The Lancet Infectious Diseases* (2020). 10.1016/S1473-3099(20)30553-3 PMC7329287 32621869

[10] Chernozhukov, V., Kasaha, H. & Schrimpf, P. Causal impact of masks, policies, behavior on early covid-19 pandemic in the us. *arXiv preprint* arXiv:2005.14168 (2020). 10.1016/j.jeconom.2020.09.003 PMC7568194 33100476

[11] Gatto M (2020). Spread and dynamics of the COVID-19 epidemic in Italy: effects of emergency containment measures. Proc. Natl. Acad. Sci..

[12] Huber, M. & Langen, H. The impact of response measures on covid-19-related hospitalization and death rates in Germany and Switzerland (2020). arXiv:2005.11278. 10.1186/s41937-020-00054-w PMC7447586 32864361

[13] Chinazzi M (2020). The effect of travel restrictions on the spread of the 2019 novel coronavirus (covid-19) outbreak. Science.

[14] Gatto M (2020). Spread and dynamics of the covid-19 epidemic in Italy: effects of emergency containment measures. Proc. Natl. Acad. Sci..

[15] Hadjidemetriou GM, Sasidharan M, Kouyialis G, Parlikad AK (2020). The impact of government measures and human mobility trend on covid-19 related deaths in the UK. Transp. Res. Interdiscip. Perspect..

[16] Chen, X. & Qiu, Z. Scenario analysis of non-pharmaceutical interventions on global covid-19 transmissions. *arXiv preprint* arXiv:2004.04529 (2020).

[17] Flaxman S (2020). Estimating the effects of non-pharmaceutical interventions on COVID-19 in Europe. Nature.

[18] Bonardi J, Gallea Q, Kalanoski D, Lalive R (2020). Fast and local: How lockdown policies affect the spread and severity of covid-19. Covid Economics, Vetted and Real-Time Papers.

[19] Hale, T., Webster, S., Petherick, A., Phillips, T. & Kira, B. Oxford covid-19 government response tracker. *Blavatnik School of Government* (2020). 10.1038/s41562-021-01079-8 33686204

[20] ECDC - European Centre for Disease Prevention and Control, The Geographic Distribution of COVID-19 cases worldwide. https://www.ecdc.europa.eu/en/publications-data/download-todays-data-geographic-distribution-covid-19-cases-worldwide (2020).

[21] MacKinnon, J. G. & Magee, L. Transforming the dependent variable in regression models. *International Economic Review* 315–339, (1990).

[22] Google, COVID-19 Community Mobility Reports. https://www.google.com/covid19/mobility/ (2020).

[23] Kleven H, Landais C, Søgaard JE (2019). Children and gender inequality: Evidence from denmark. Am. Econ. J. Appl. Econ..

[24] Kermack WO, McKendrick AG (1927). A contribution to the mathematical theory of epidemics. Proceedings of the royal society of london. Series A, Containing papers of a mathematical and physical character.

[25] Abraham, S. & Sun, L. Estimating dynamic treatment effects in event studies with heterogeneous treatment effects. *Available at SSRN* **3158747**, (2018).

[26] Borusyak, K. & Jaravel, X. Revisiting event study designs. *Available at SSRN* **2826228**, (2017).

[27] Correia, S. Linear models with high-dimensional fixed effects: an efficient and feasible estimator. Tech. Rep. (2016). Working Paper.

[28] Lozzi, G. *et al.* Covid-19 and urban mobility: impacts and perspectives—rapid-response briefing. https://www.europarl.europa.eu/RegData/etudes/IDAN/2020/652213/IPOL_IDA(2020)652213_EN.pdf. Accessed 21 Dec 2020.

[29] Leung NHL (2020). Respiratory virus shedding in exhaled breath and efficacy of face masks. Nat. Med..

