# Supplementary material for: Estimating worldwide effects of non-pharmaceutical interventions on COVID-19 incidence and population mobility patterns using a multiple-event study
Source: Sci Rep. 2021 Jan 21;11:1972. doi: 10.1038/s41598-021-81442-x (PMC7820317; doi:10.1038/s41598-021-81442-x)
Supplement: Supplementary file 1 — Supplementary Figures. [file 41598_2021_81442_MOESM1_ESM.pdf]

Estimating worldwide effects of non-pharmaceutical interventions on COVID-19 incidence and population mobility patterns using a multiple-event study

Supplementary Information

Nikolaos Askitas*

IZA - Institute of Labor Economics and CESifo

Konstantinos Tatsiramos

University of Luxembourg, LISER, IZA and CESifo

Bertrand Verheyden

Luxembourg Institute of Socio-Economic Research (LISER)

* Corresponding author.

A Appendix - Figures

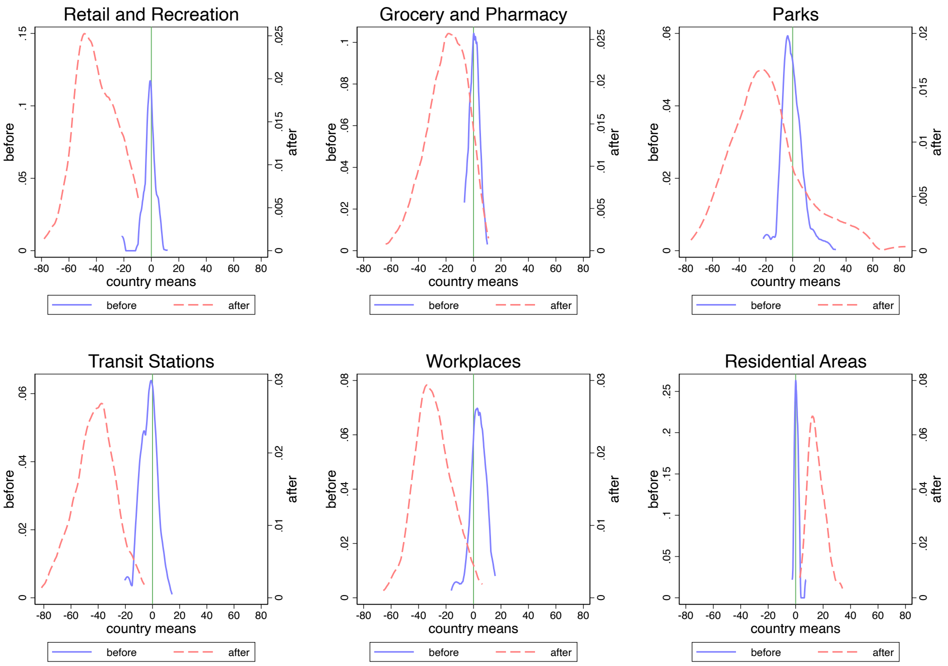

Figure A1: Summary statistics: mobility types

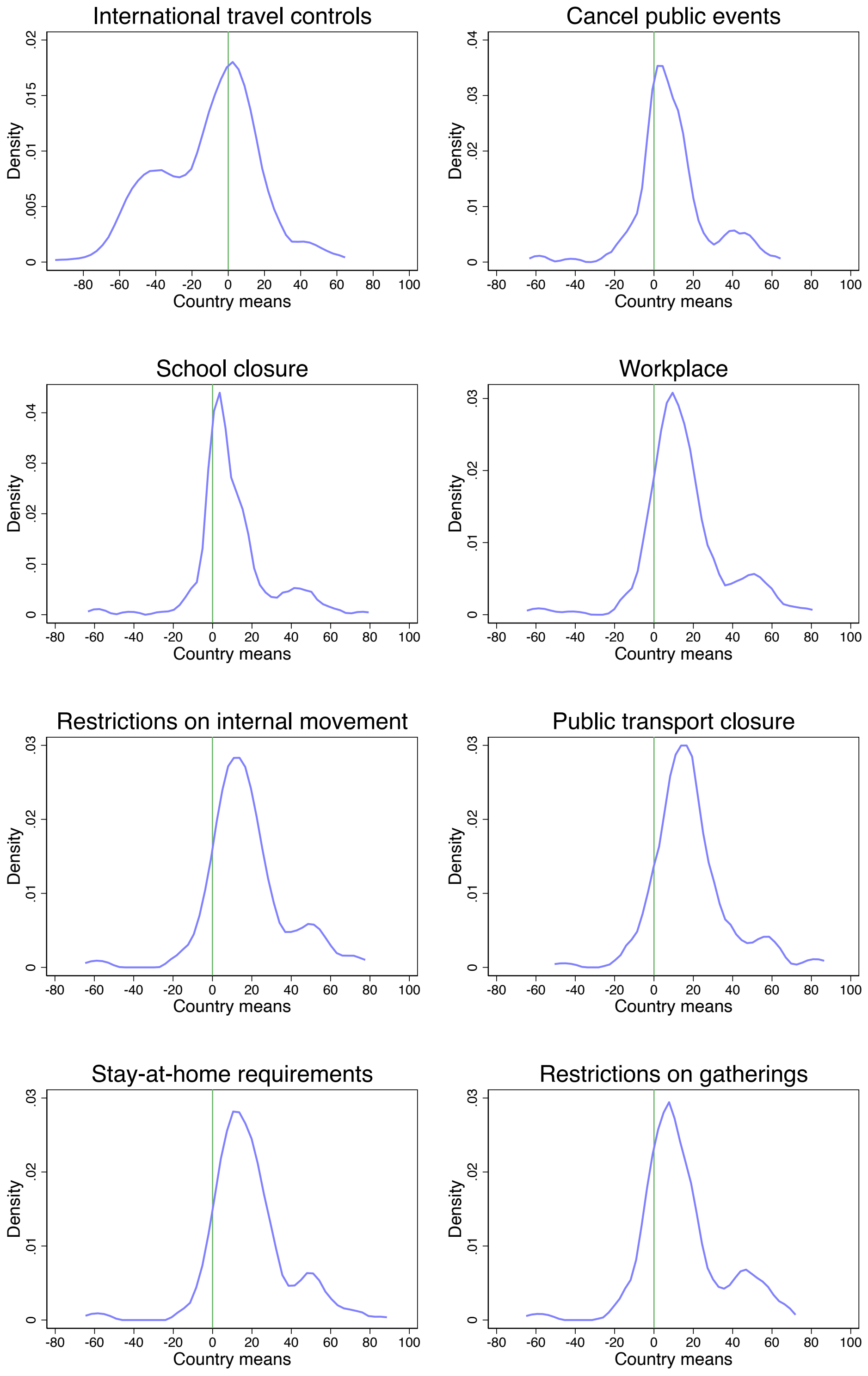

Figure A2: Summary statistics: policies

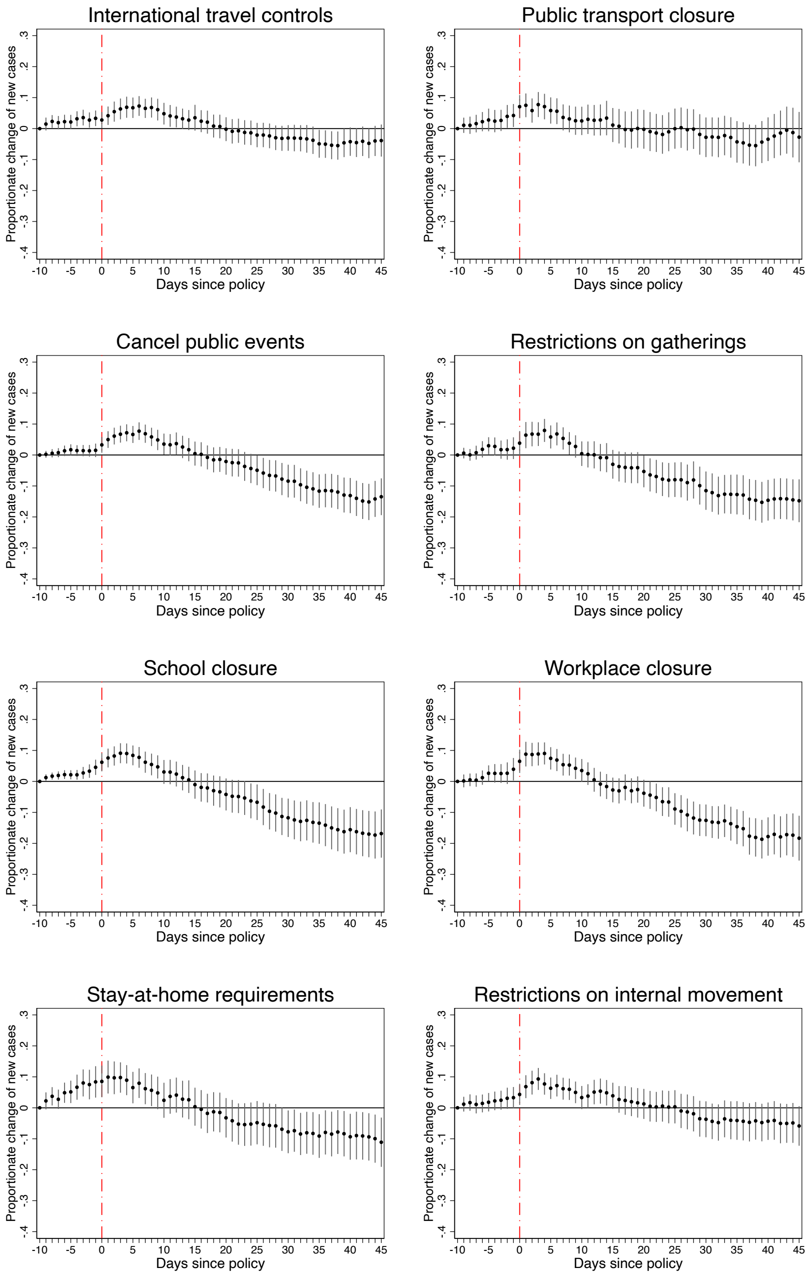

Note: Data from Hale et al. (2020), European CDC and own calculations

Figure A3: Effects of lockdown policies on **COVID-19** confirmed new cases (3-day moving average, in logs) without concurrent policy controls.

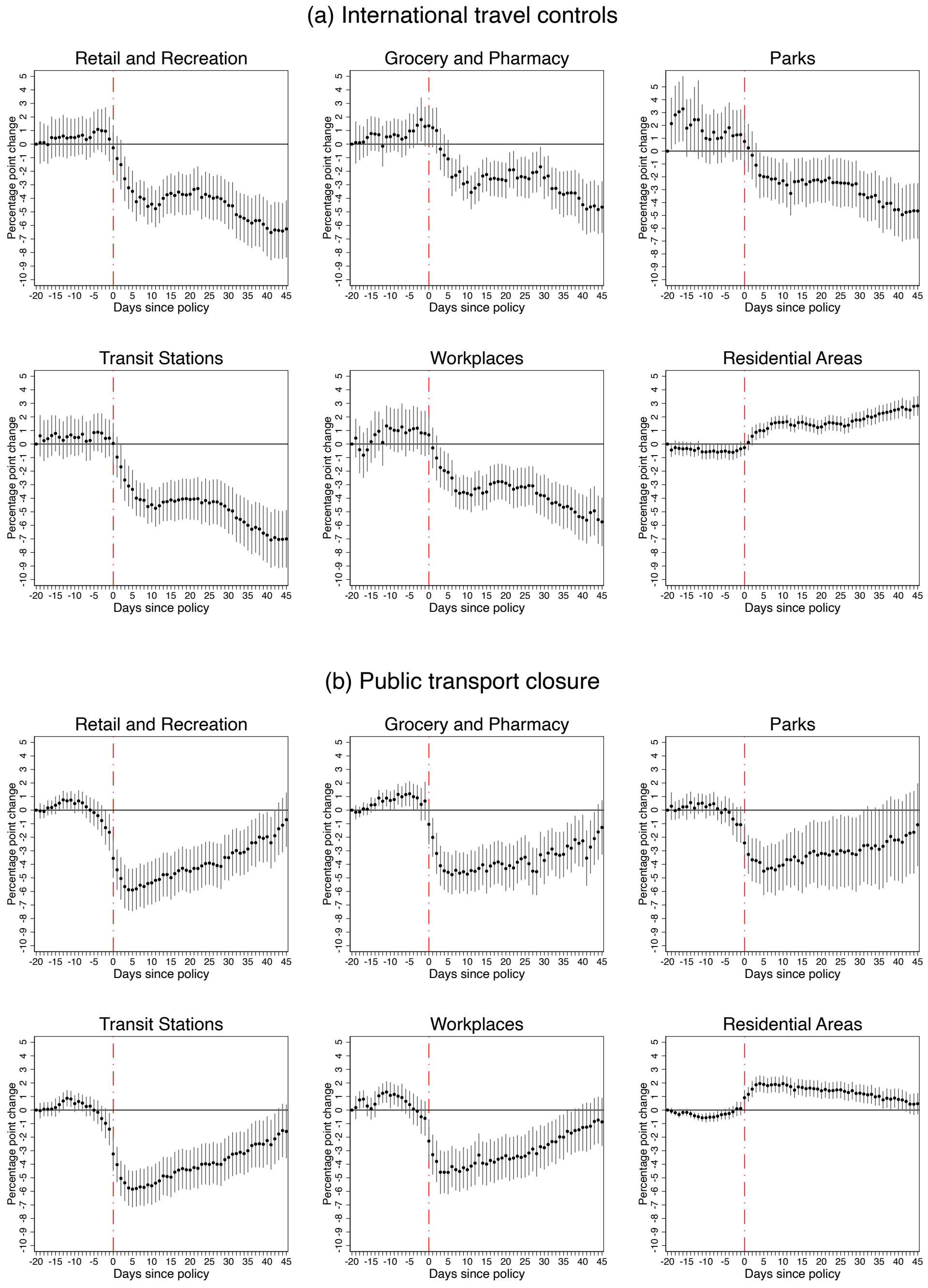

Note: Data from Hale et al. (2020), Google Community Mobility Reports and own calculations

Figure A4: Effects of **international travel controls** (panel a) and **public transportation closure** (panel b) on Google mobility patterns without concurrent policy controls.

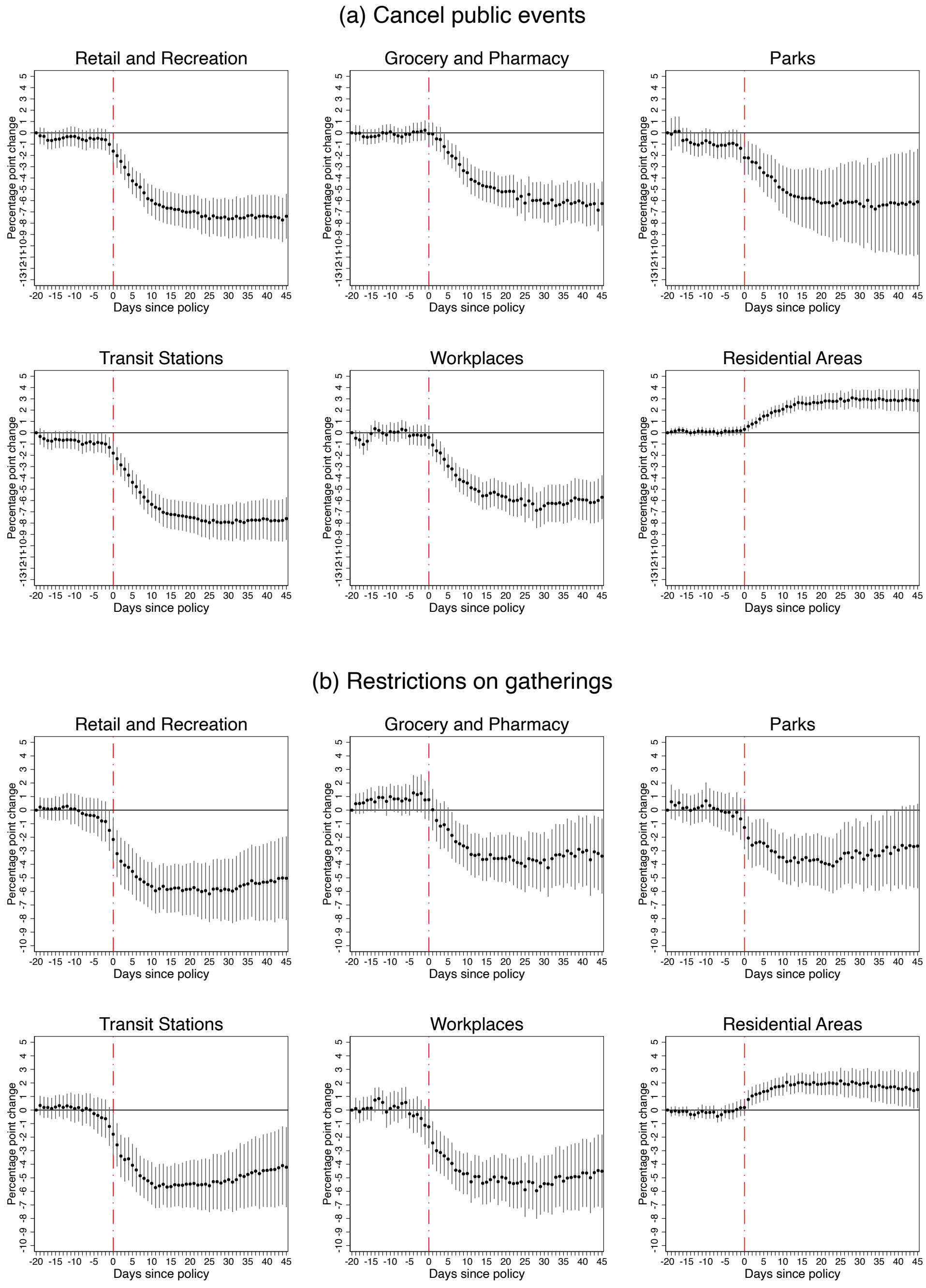

Note: Data from Hale et al. (2020), Google Community Mobility Reports and own calculations

Figure A5: Effects of **public events cancellations** (panel a) and **restrictions on gatherings** (panel b) on Google mobility patterns without concurrent policy controls.

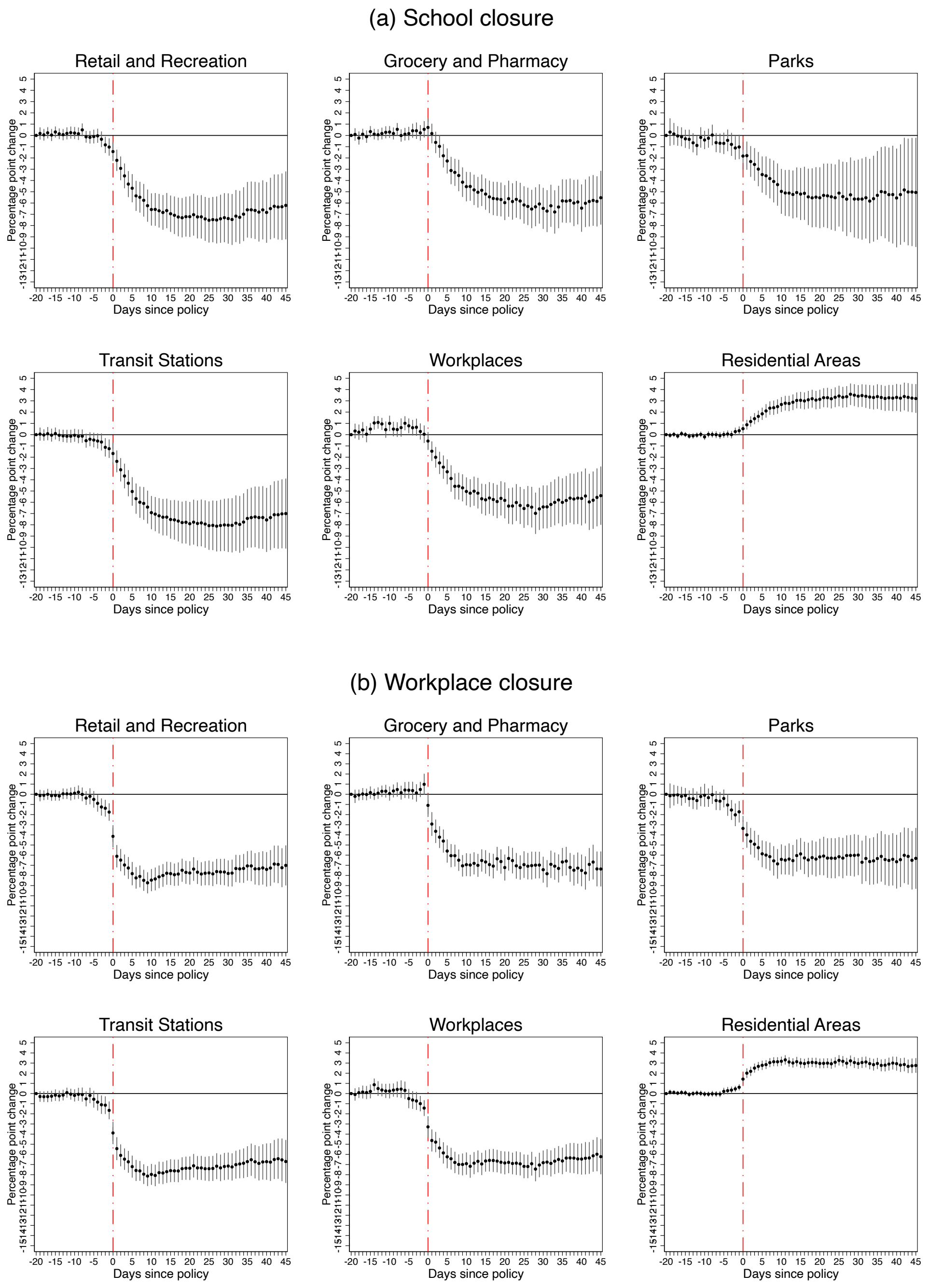

Note: Data from Hale et al. (2020), Google Community Mobility Reports and own calculations

Figure A6: Effects of **school** (panel a) and **workplace** (panel b) closures on Google mobility patterns without concurrent policy controls.

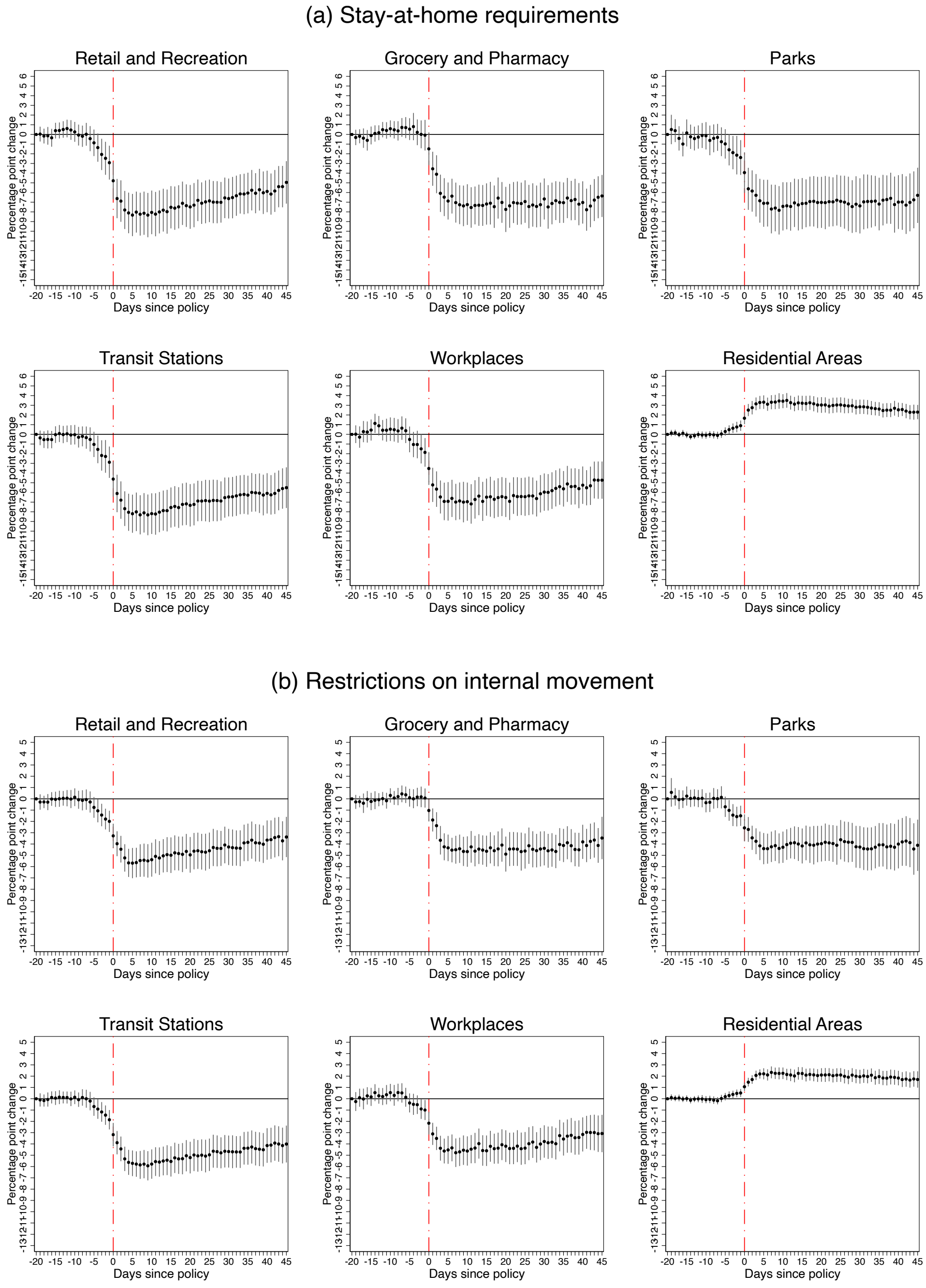

Note: Data from Hale et al. (2020), Google Community Mobility Reports and own calculations

Figure A7: Effects of **stay-at-home requirements** (panel a) and **restrictions on internal mobility** (panel b) on Google mobility patterns without concurrent policy controls.
